# Validity and reliability of medication adherence scale in FMF (adult version)

**DOI:** 10.1186/1546-0096-12-S1-P243

**Published:** 2014-09-17

**Authors:** Berna Eren Fidanci, Sirzat Yesilkaya, Cengizhan Acikel, Aslan Ozden, Dogan Simsek, Fatih Yildiz, Bunyamin Kisacik, Mehmet Sayarlıoglu, Servet Akar, Soner Senel, Mehmet Tunca, Sule Yavuz, Abdurrahman Tufan, Afig Berdeli, Ahmet Onat, Ahmet Gul, Berna Goker, Timucin Kasifoglu, Haner Direskeneli, Sukran Erten, Gul Ozcelik, Faysal Gok, Seza Ozen, Erkan Demirkaya

**Affiliations:** 1 FAVOR, FMF Arthritis Vasculitis and Orphan Disease Research in Paediatric Rheumatology, Ankara, Turkey; 2 Turkish FMF Study Group, Ankara, Turkey

## Introduction

MASIF (Medication Adherence Scale in FMF) is an instrument designed to measure adherence to treatment in children with Familial Mediterranean Fever (FMF). We have developed this scale for children with FMF and found valid and reliable.

## Objectives

In this study, it was aimed to assess the validity and reliability of this adherence scale for medical treatment in adult FMF patients.

## Methods

This study is multicentre and 14 centers participated to the study. Patients with FMF using medication at least for 6 months and accepted to participate constituted the sample of the study. Besides “Medication Adherence Scale in FMF Patients (MASIF)”, “Data collection forms about the sociodemographic and medical information (demographic, clinical and laboratory findings) of patients”, and “Morisky Medication Adherence Scale (MMAS)” were used as data collection instruments.

## Results

There were 133 patients with FMF enrolled for the validation of the study. The median age of the patients (n=133) was 28.60 years (min.18.12-max.71.34) and 52.6% of them were female. The median number of the attack frequency was 13.50 (min. 0-max 99) in a year and 57.9% of the participants had irregular attacks. For internal consistency, Cronbach’s alpha was 0,764 for MASIF adult version. Also, there was a positive and significant correlation between test and retest score (t=0.971; p=0.340). Morisky was used as gold standard. For the "criterion validity" the correlation with Morisky and MASIF was evaluated (r=0.530, p=0.000) and for the "structure" validity, factor analyzes and Kaiser-Meyer-Olkin tests were performed.

## Conclusion

Approximately 10-15% of patients with FMF are non-responders but it was claimed that in fact they are non-compliers that causes these patients receive unnecessary biologic agent treatment procedures, which are hazardous as well as expensive. This scale will provide assessment and follow up of adherence to treatment patients and determine whether the patient is non-responders or non-compliers. It may help to determine the non-compliance and prevent unnecessary and expensive biologic agents.

## Disclosure of interest

None declared.

